# The Recombinant Oncolytic Virus VV-GMCSF-Lact and Chemotherapy Drugs against Human Glioma

**DOI:** 10.3390/ijms25084244

**Published:** 2024-04-11

**Authors:** Natalia Vasileva, Alisa Ageenko, Arina Byvakina, Aleksandra Sen’kova, Galina Kochneva, Sergey Mishinov, Vladimir Richter, Elena Kuligina

**Affiliations:** 1 Institute of Chemical Biology and Fundamental Medicine Siberian Branch of the Russian Academy of Sciences, Akad. Lavrentiev Ave, 8, Novosibirsk 630090, Novosibirsk Region, Russia; a.ageenko@alumni.nsu.ru (A.A.); mytrilliangalaxy@gmail.com (A.B.); alsenko@mail.ru (A.S.); richter@niboch.nsc.ru (V.R.); kuligina@niboch.nsc.ru (E.K.); 2 “Oncostar” LLC, Inzhenernaya Street 23, Novosibirsk 630090, Novosibirsk Region, Russia; 3 The State Research Center of Virology and Biotechnology “VECTOR”, Koltsovo 630559, Novosibirsk Region, Russia; kochneva@vector.nsc.ru; 4 Novosibirsk Research Institute of Traumatology and Orthopedics n.a. Ya.L. Tsivyan, Frunze Street 17, Novosibirsk 630091, Novosibirsk Region, Russia; smishinov@yandex.ru

**Keywords:** glioma, oncolytic virus, vaccinia virus, VV-GMCSF-Lact, temozolomide

## Abstract

Virotherapy is one of the perspective technologies in the treatment of malignant neoplasms. Previously, we have developed oncolytic vaccinia virus VV-GMCSF-Lact and its high cytotoxic activity and antitumor efficacy against glioma was shown. In this work, using immortalized and patient-derived cells with different sensitivity to VV-GMCSF-Lact, we evaluated the cytotoxic effect of chemotherapy agents. Additionally, we studied the combination of VV-GMCSF-Lact with temozolomide which is the most preferred drug for glioma treatment. Experimental results indicate that first adding temozolomide and then the virus to the cells is inherently more efficient than dosing it in the reverse order. Testing these regimens in the U87 MG xenograft glioblastoma model confirmed this effect, as assessed by tumor growth inhibition index and histological analysis. Moreover, VV-GMCSF-Lact as monotherapy is more effective against U87 MG glioblastoma xenografts comparing temozolomide.

## 1. Introduction

Glioblastoma remains one of the most aggressive types of tumors and is difficult to treat. In addition, since the inclusion of temozolomide in the standard regimen in 2005 [1], no significant progress has been made in developing new approaches that would be included in the standard therapy. Thus, the search for more effective methods of therapy for malignant gliomas is an important task of current research.

Temozolomide (TMZ) is an imidazotetrazine derivative of the alkylating agent dacarbazine, at physiological pH, the drug is hydrolyzed to a short-lived active component (3-methyl-(triazen-1-yl)imidazole-4-carboximide (MTIC)). This compound is known to introduce methyl groups at the N7 and O6 positions of guanines and the N3 position of adenines in genomic DNA, which ultimately leads to cell cycle arrest and apoptosis [2]. The therapeutic effectiveness of temozolomide depends on the activity of MGMT in tumor cells. MGMT reduces alkylated DNA adducts in a single step. O6-MeG during DNA replication pairs with thymine instead of cytosine, which leads to cell death at low levels of MGMT. In turn, MMR (mismatch repair system) recognizes only unpaired thymine in the daughter strand, while O6-MeG remains in the matrix. Further cycles of thymine reinsertion and excision lead to persistent DNA strand breaks, replication fork collapse, and replication arrest [3]. Since both methylation processes of the *MGMT* promoter and MMR function can be impaired in glioblastoma cells, the therapeutic effect of temozolomide may be limited.

Another chemotherapeutic method for the treatment of glioblastoma is the PCV regimen. PCV is a combination of procarbazine, lomustine (CCNU), and vincristine [4]. Lomustine, a nitrosourea derivative, exhibits both alkylating and chlorethylating properties. The resulting O6ClEtG adduct is chemically unstable and undergoes intramolecular rearrangement with the formation of interchain cross-links N1-O6-ethenoguanine and, finally, N1-guanine-N3-cytosine [5]. Since O6ClEtG is also a substrate for MGMT, the level of this enzyme determines the effectiveness of lomustine therapy. However, the combined action of procarbazine, another alkylating agent, and a nitrosourea derivative, is known to reduce MGMT activity [6].

Vincristine belongs to a group of alkaloids obtained from a common flowering herb, the periwinkle plant (Vinca rosea Linn) [7]. Vincristine binds tubulin and inhibits microtubule formation, resulting in mitotic arrest. In addition, vincristine inhibits the synthesis of proteins and nucleic acids, blocking the utilization of glutamic acid [8]. The PCV regimen in combination with radiotherapy achieves an antineoplastic effect, but this combination of drugs has a pronounced neurotoxicity that exceeds the neurotoxicity of each of the components [9].

At the same time, one of the most actively developing antitumor approaches for today is therapy with oncolytic viruses. Virotherapy is an immunotherapeutic approach based on virus-mediated tumor cell death and induction of the immune response [10]. According to clinicaltrials.gov, various oncolytic viruses are currently undergoing clinical trials as potential agents for the treatment of brain tumors, including glioblastoma. Recently, recombinant oncolytic herpes simplex virus type 1 G47Δ became the first approved oncolytic virus in the world for the treatment of malignant gliomas in Japan [11]. Taking into account this significant achievement, it is necessary to further develop research on oncolytic viruses for cancer treatment.

Despite the obvious advantages, virotherapy has certain disadvantages that must be considered when developing therapeutic scheme with the use of oncolytic viruses. Among the factors limiting the effectiveness of virotherapy, one can distinguish the following: the ability of a tumor to avoid an immune response, even with its additional induction due to the spread of viral particles; the viscosity of the extracellular matrix which can affect the spread of viral particles from cell to cell; the production of virus-neutralizing antibodies that reduce the effectiveness of virotherapy with repeated injections; and others. To overcome these limitations, oncolytic viruses can be used in combination with various antitumor approaches, the disadvantages of which, in turn, can be compensated by virotherapy. Since chemotherapy is the main treatment, it is therefore important to evaluate the combination of the virus with chemotherapy at the stage of preclinical studies. At the same time, oncolytic viruses can affect DNA repair mechanisms, increasing the cytotoxic effect of chemotherapy drugs [12].

VV-GMCSF-Lact was previously developed by the team of the ICBFM SB RAS and the SSC VB Vector on the basis of the vaccinia virus L-IVP strain. VV-GMCSF-Lact carries deletions of fragments of the viral thymidine kinase and growth factor genes, in the regions of which genes of the human GM-CSF and the apoptosis-inducing protein lactaptin are inserted, respectively [13]. GM-CSF induces an antitumor immune response by recruiting granulocytes and macrophages, and also stimulates the differentiation of dendritic cells [14]. Lactaptin is a proteolytic fragment of kappa-casein in human milk and has oncotoxic activity against human cancer cells inducing cancer cell apoptosis via the mitochondrial pathway [15]. VV-GMCSF-Lact is currently undergoing clinical trials as a treatment for breast cancer, including triple negative (NCT05376527). In addition, the cytotoxic activity and antitumor efficacy of the virus against human glioblastoma have already been shown [16].

In this work, we assessed the sensitivity of cells of immortalized U87 MG and U343 MG lines and patient-derived BR2.20 and BR5.21 cultures of human gliomas to the action of chemotherapeutic agents (temozolomide, lomustine, procarbazine, and vincristine). According to the data obtained, the studied cultures have different sensitivity to chemotherapy drugs. In addition, all studied cultures are characterized by resistance to procarbazine.

Also, we evaluated the effectiveness of the combined action of VV-GMCSF-Lact and temozolomide against cells of immortalized and patient-derived glioblastoma cultures. We have shown that the cells of the studied cultures have different sensitivity to the combination of the virus and temozolomide. At the same time, VV-GMCSF-Lact probably inhibits the action of temozolomide when a chemotherapeutic agent is introduced into the regimen after cell treatment with the virus. While adding temozolomide to cells first, and then the virus, a synergistic or additive effect develops for cells of most cultures studied. Using U87 MG xenograft glioblastoma models, the combined action of VV-GMCSF-Lact and TMZ was evaluated. We have demonstrated that VV-GMCSF-Lact, both in monotherapy and in combination with TMZ, is highly effective and, moreover, significantly more effective than TMZ alone.

Based on the data received, we propose a therapeutic scheme for the combined use of temozolomide and VV-GMCSF-Lact, in which the chemotherapeutic drug should be used no earlier than 7–8 days after the virus use.

## 2. Results

### 2.1. VV-GMCSF-Lact Sensitivity of Glioma Cells

Previously, we analyzed the VV-GMCSF-Lact cytotoxic effect on different glioma cells, including immortalized U87 MG and U343 MG glioblastoma cells that were also used in this work [16].

To estimate the VV-GMCSF-Lact cytotoxic effect, we used patient-derived cell cultures BR2.20 (diffuse astrocytoma, grade 3) and BR5.21 (anaplastic astrocytoma, grade 3), obtained from patient tumor samples. According to the data, obtained cells of various cultures have different sensitivity to VV-GMCSF-Lact (Table 1). The sensitivity of cell cultures used in this work to the virus decreases in the series BR2.20 > U343 MG > U87 MG > BR5.21.

### 2.2. Sensitivity of Glioma Cells to Temozolomide, Lomustine, Procarbazine, and Vincristine

Temozolomide (TMZ) is an imidazotetrazine second-generation alkylating agent which is the main drug in the adjuvant glioma treatment [1]. The PCV regimen (a combination of procarbazine, lomustine, and vincristine) is another treatment option for adult glioma, but it has significant toxicity and is rarely used [4].

To evaluate the cytotoxic activity of chemotherapy agents, U87 MG, U343 MG, BR2.20, and BR5.21 cells were used. All the studied cultures are characterized by resistance to procarbazine in the concentration range from 0.09 mg/mL to 1 mg/mL. The U343 MG cells are the most sensitive to the other three chemotherapy drugs studied—lomustine, vincristine, and temozolomide. The most resistant to temozolomide are U87 MG cells, and the most resistant to vincristine and lomustine are BR5.21 cells (Table 2).

### 2.3. Combined Action of VV-GMCSF-Lact and Temozolomide In Vitro

Since the “gold standard” in the first line of treatment for patients with glioma is the maximum possible tumor resection followed by chemotherapy with TMZ, the effect of VV-GMCSF-Lact in combination with TMZ against studied glioma cells was analyzed. Based on the hypothesis that VV-GMCSF-Lact will be used to treat the resection cavity in clinical trials for brain tumor treatment, we conclude that TMZ will be introduced into the treatment regimen after the virus. Therefore, we performed in vitro studies of this combination according to a similar protocol.

The cells were treated with the virus (at a multiplicity of infection equal to the CD50 individual for each culture (the dose at which 50% of cells die)). TMZ was added to the cells at various concentrations, which were individual for each culture. Concentrations were determined based on the sensitivity of cells of a particular culture to the drug. For the test, concentrations of the drug were chosen at less than CD50, CD50, and more than CD50. TMZ was added at different times after the virus administration. Since no improvement in cytotoxic effect was observed at intervals of 12, 24, 36, and 48 h, for comparison, we consider only the data obtained when TMZ was added to cells after 24 h and 60 h of incubation with the virus.

**Figure 1:**
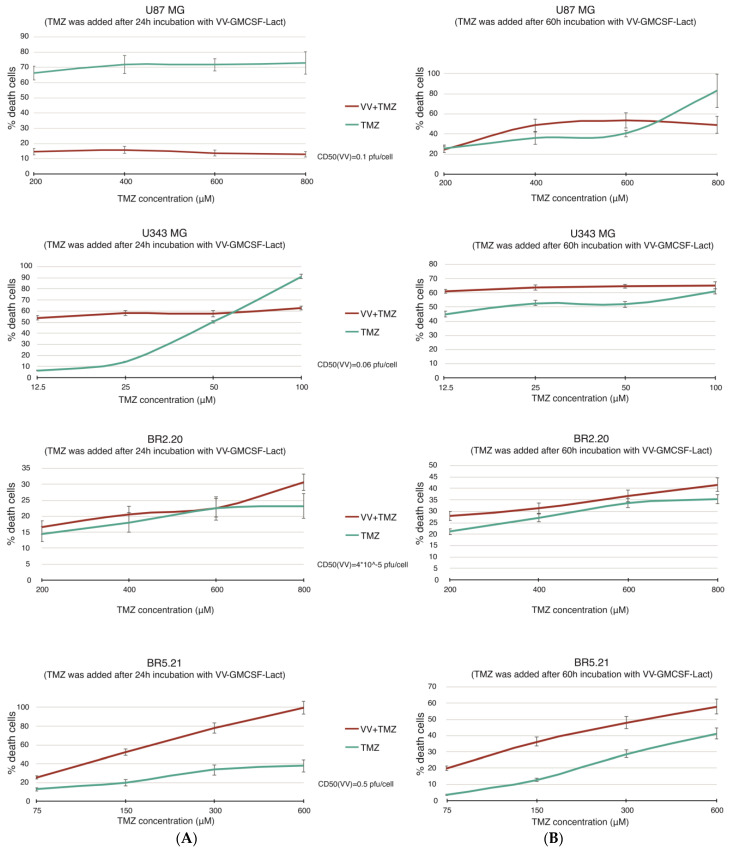
Changes in the viability of human glioma cells under the action of VV-GMCSF-Lact followed by temozolomide addition. (**A**)—TMZ was added to cells after 24 h incubation with VV-GMCSF-Lact; (**B**)—TMZ was added to cells after 60 h incubation with VV-GMCSF-Lact. CD50(VV)—multiplicity of virus infection, in which 50% of cells die; VV—VV-GMCSF-Lact; TMZ—temozolomide.

Based on the results obtained (Figure 1), it can be concluded that the most resistant to VV-GMCSF-Lact culture BR5.21, when incubated with the virus and subsequent addition of TMZ at various concentrations, is characterized by greater sensitivity to such a combination. The rest of the studied cultures demonstrate the resistance to the virus–temozolomide combination when the chemotherapeutic agent is added 24 h after the virus. However, when TMZ is added 60 h after VV-GMCSF-Lact, greater effects are observed for all cell cultures, except for U87 MG. The combination index (CI) for U87 MG is 1.6, and the CI for other studied cultures is from 0.5 to 0.7 for the highest TMZ concentrations (Figure 1).

It will also be interesting to evaluate the effect of the combination when TMZ is added first to the cells and then to the virus. For this, cells were treated with TMZ at various concentrations, individual for each culture, and after 24 h from the start of incubation with TMZ, the virus was added to the cells at a dose equal to CD50 for each culture under study. The total time of cell incubation with the drugs was 72 h at 37 °C in an atmosphere of 5% CO_2_.

According to this scheme, the obtained data indicate that the combined action of drugs is characterized by the additive effect for studied glioma cells at the points for the highest TMZ concentrations (CI are from 1.0 to 1.4) (Figure S1).

### 2.4. Combined Action of VV-GMCSF-Lact and Temozolomide In Vivo

To estimate VV-GMCSF-Lact and TMZ antitumor efficacy, a subcutaneous xenograft model was used. U87 MG cells, subcutaneously transplanted into SCID mice, were utilized as the established model. Tumor-bearing mice were treated as described in Table 3.

**Figure 2:**
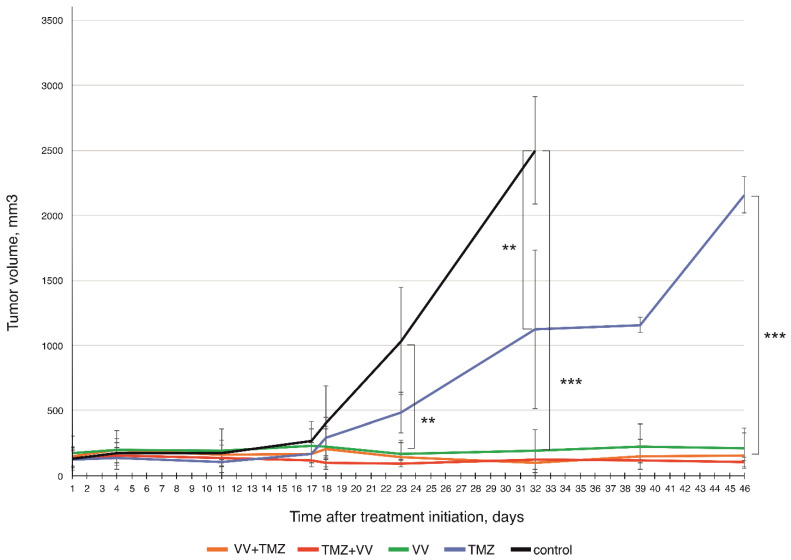
Antitumor efficacy of VV-GMCSF-Lact, temozolomide, and their combination against subcutaneously transplanted U87 MG glioblastoma. Data are presented as mean ± SD (*n* = 6). **—*p* ≤ 0.01, ***—*p* ≤ 0.001. Statistical analysis was performed using the Mann–Whitney U-test.

The obtained results indicate that VV-GMCSF-Lact, both in monotherapy and in combination with TMZ, regardless of the order of drug administration, has high antitumor efficacy (Figure 2). Thus, significant differences in tumor volumes of the “VV-GMCSF-Lact”, “VV-GMCSF-Lact+TMZ”, and “TMZ+VV-GMCSF-Lact” groups compared to the control group were shown already on the 23rd day from the start of treatment (*p* ≤ 0.01). At the same time, significant differences in tumor volumes in the control group and the group receiving TMZ injections were shown only by 32 days from the start of treatment. The tumor volumes in the “VV-GMCSF-Lact”, “VV-GMCSF-Lact+TMZ”, and “TMZ+VV-GMCSF-Lact” groups were significantly less than in the TMZ group, starting from the 32nd day of the experiment, by the end of the experiment (*p* ≤ 0.001).

The tumor growth inhibition indexes (TGI) for the “VV-GMCSF-Lact”, “VV-GMCSF-Lact+TMZ”, and “TMZ+VV-GMCSF-Lact” groups did not differ and were 93%, which indicates that all studied treatment regimens are possible for use. At the same time, when using TMZ in monotherapy, the TGI index was only 33% (Table 4).

**Figure 3:**
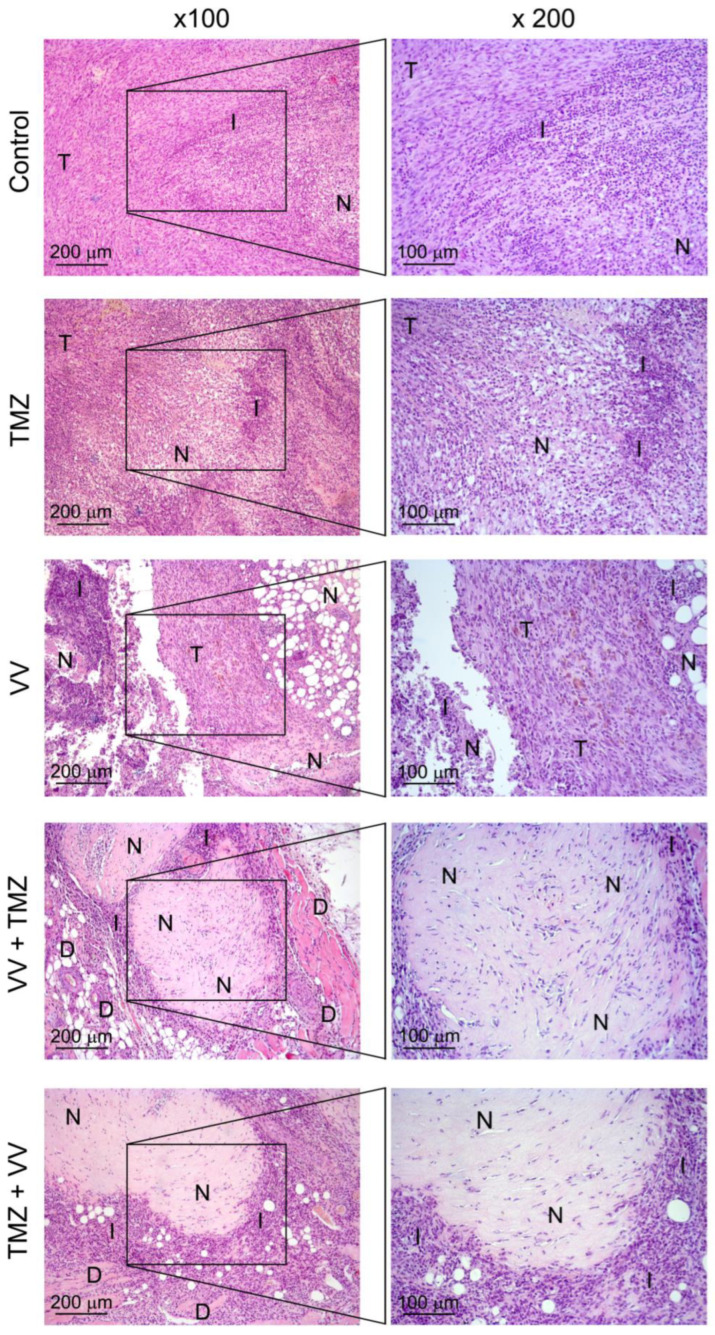
Structural changes in glioblastoma U87 MG, subcutaneously transplanted into SCID mice, without treatment (control), and after administration of temozolomide (TMZ, intraperitoneal administration) and VV-GMCSF-Lact (VV, intratumoral administration), and also with combination therapy (VV+TMZ and TMZ+VV). Hematoxylin and eosin staining. Original magnification ×100 (left panel) and ×200 (right panel) T—unchanged tumor tissue, N—foci of necrosis, I—areas of infiltration with granulocytes, D—remnants of epidermis-dermis complex and subcutaneous tissue.

Moreover, the study of the characteristics of the antitumor activity of temozolomide, a viral drug, and their combination was carried out by histological assessment of the structural organization of tumor nodes of glioblastoma U87 MG without treatment and after administration of TMZ, a viral drug (VV-GMCSF-Lact, hereinafter VV), and their combination (Figure 3, Table 5).

The tumor node in the control group has a round shape with clear boundaries and is represented by spindle-shaped cells with a thin rim of cytoplasm and hyperchromic nuclei. The tumor tissue contains a large number of deformed thin-walled vessels and a moderate number of mitoses (5.5 ± 0.7 per unit of test area). In the central part of the tumor node, a focus of necrosis is determined, occupying 52.1 ± 9.5% of the entire section area, the formation of which may be associated with insufficient blood supply to the tumor by newly formed vessels with an imperfect structure. At the border between necrosis and unchanged tumor tissue, an accumulation of a large number of granulocytes, which is of a reactive nature, is determined.

With intraperitoneal administration of TMZ, pronounced destructive changes were observed in the tumor node, represented by massive foci of necrosis, abundantly infiltrated with granulocytes and constituting 62.5 ± 12% of the section area. A decrease in the proliferative activity of the tumor was also revealed; the numerical density of mitoses was 3.6 ± 0.6 per unit of test area.

Intratumoral administration of the VV also caused the development of destructive changes in the tumor node, which had some morphological features, namely, massive foci of necrosis contained a large number of cystic structures, the formation of which may be associated with the death of tumor cells and destruction of the extracellular matrix under the influence of the virus. The focus of necrosis, completely infiltrated with granulocytes, was also determined separately. Necrotic changes in the tumor tissue accounted for 74.5 ± 9.8% of total section area. When counting mitoses, a significant decrease in their numerical density was revealed by 9.2 times compared to the control.

Combination therapy with VV followed by TMZ caused massive cell migration into the tumor tissue. Histological examination of tumor nodes revealed foci of completely altered tumor tissue, infiltrated with granulocytes and separated from each other by the layers of tumor cells, which were also diffusely infiltrated with granulocytes. Foci of destruction in this case occupied 93.6 ± 3.6% of the entire section area.

During combination therapy with TMZ followed by the administration of VV, the tumor node was completely represented by foci of necrotic decay, abundantly infiltrated with granulocytes, along the periphery of which only remnants of tumor tissue were found. However, during a morphometric study, the degree of destruction was somewhat less pronounced than in the previous group; foci of necrosis occupied 66.8 ± 2.5% of the section area.

With two combination treatment regimens, no mitotic events were detected in the tumor tissue (Table 5).

## 3. Discussion

The main glioma treatment today is based on a combination of maximum surgical resection, radiotherapy, and chemotherapy. Tumor resection reduces the mass effect and provides tissue for subsequent histological analysis and molecular characterization [17]. However, even with maximal surgical resection, relapses occur, since glioma cells are able to migrate to distant parts of the brain and sometimes form extracranial metastases [18,19].

The standard adjuvant chemotherapy for the glioma treatment is temozolomide, approved for clinical use in 2005 [1]. But cancer cells are able to acquire resistance to chemotherapy by alkylating agents. Since both methylation of the *MGMT* promoter and MMR function can be disrupted in glioma cells, the therapeutic effect of temozolomide is often limited [20]. For this reason, other chemotherapy drugs can be used, for example, the PCV regimen or procarbazine, lomustine or vincristine alone, or in combination with TMZ. However, these drugs also have serious toxic effects and consequences. Procarbazine can cause granulomatous hepatitis. CCNU (lomustine) and vincristine also have hepatotoxicity. Among others, various types of neuropathy and paralysis of the eyes or vocal cords are observed [21,22,23].

First of all, in this study, we evaluated the oncotoxic effect of chemotherapeutic agents such as temozolomide, vincristine, procarbazine, and lomustine against cells of immortalized U87 MG and U343 MG and patient-derived BR2.20 and BR5.21 glioma cell cultures. We showed that cells have different sensitivity to the action of drugs, which once again confirms the feasibility of a precision approach to the treatment of gliomas, since in different tumors, the pathways responsible for resistance to chemotherapy can be activated differently.

In addition to the chemotherapy drugs already used in the clinic, researchers are actively developing other antitumor agents that can improve the effectiveness of therapy and the quality of life of patients with gliomas. A promising immunotherapeutic drug for the treatment of glioblastoma is, in particular, a double recombinant vaccinia virus VV-GMCSF-Lact [13]. To date, this oncolytic virus has been tested in phase I clinical trials as drug for breast cancer therapy (NCT05376527).

Since chemotherapy drugs recognized in clinical practice are still widely used for tumor therapy, one of the ways to introduce the OV’s into practice is combination therapy. This often makes it possible to negate the paths that inhibit the effect of a particular agent.

In this work, we also assessed the cytotoxic activity of the oncolytic virus VV-GMCSF-Lact and the chemotherapy drug TMZ against cells of immortalized human glioblastoma lines and patient-derived glioma cultures. According to the data obtained, cells have different sensitivity to the action of VV-GMCSF-Lact and TMZ. In addition, the combined effect of VV-GMCSF-Lact with TMZ was evaluated against cells of the studied glioma cultures.

There is some research which considers the combination of TMZ and oncolytic viruses against various cancer types. Some publications report that virotherapy and TMZ chemotherapy have a synergistic effect [24,25,26]. Moreover, Ryuichi Kanai and colleagues have shown that the herpes simplex virus G47Δ in combination with TMZ is effective against glioma cancer stem cells [25]. Studies of the same G47Δ virus, but armed with an insertion encoding mouse interleukin 12 (G47Δ-IL12), demonstrate a synergistic effect on mouse glioma cancer stem cells in vitro. However, the data obtained on the in vivo model indicate a decrease and even no effect [27]. But data on the combined action of vaccinia virus and alkylating agents could not be found.

In our data, the BR5.21 culture, which is the most resistant to VV-GMCSF-Lact, is characterized by the highest sensitivity to the virus–temozolomide combination. Cells of other cultures show resistance to the combination of drugs when the chemotherapeutic agent is added 24 h after exposure to the virus. When temozolomide is added after 60 h, a synergistic or additive effect is observed for all cultures except U87MG. Possibly, virus and TMZ can interfere with each other’s activity, so dividing the administration of drugs by time, a greater effectiveness of the combined action can be observed. There are different mechanisms of resistance of glial cells to temozolomide, some of these mechanisms may be mediated by viral replication in cells. For example, the genotoxic agent TMZ is known to predominantly induce p53-dependent apoptosis [28]. At the same time, the vaccinia virus B1 protein phosphorylates p53, mediating its degradation [29]. The B12 protein of the same virus is also able to interact with p53 and p21, thus interfering with cell cycle arrest and the induction of apoptotic cascades [30,31]. On the other hand, during a viral infection, the functioning of some cellular molecules may change, thereby causing responses such as the occurrence of an antagonistic effect of the combination with temozolomide. For example, it has been shown that in the early stages of infection, the vaccinia virus promotes the activation of cytoplasmic ATR kinase (Ataxia telangiectasia and Rad3-related kinase) [31], which is one of the key molecules in the induction of DNA repair [32]. At the same time, in glioma cells during temozolomide therapy, the MMR pathway plays an important role in resistance to the chemotherapy drug. The MutSα and MutLα complexes localize in O6MeG:T mismatches and recruit ATR [33]. As a result, either DNA repair or cell death occur if the damage cannot be repaired. Thus, the vaccinia virus may enhance the effect of resistance mechanisms of glioma cells to temozolomide.

Secondly, we have shown that the combined action of temozolomide–virus has an additive effect for all cultures. Perhaps the lack of synergy action we observed is due to the alkylating effect of TMZ which reduces the efficiency of producing DNA molecules for the assembly of new viral particles. Moreover, TMZ can also alkylate viral DNA and viral replication decreases for this reason. In addition, the reduction in the effectiveness of the virus and TMZ combination may depend on the individual biological characteristics of the cells.

The data we have obtained in in vitro models allow us to suggest a possible scheme for the use of such a combination in an in vivo model and subsequent clinical trials. The results of pharmacokinetic studies of antitumor viral drugs based on the vaccinia virus should also be taken into consideration, indicating that there are two peaks in the detection of the virus in the blood of patients. This is due to the ability of the virus to replicate in cancer cells and “supply” mature viral particles into the bloodstream. It has been shown that when injected into the organism (intratumorally or intravenously), the virus is detected in the blood 5–30 min and on the 5–7th day after the injection [34,35]. Moreover, the most promising use of VV-GMCSF-Lact in the clinic involves treating the resection field after removal of the bulk of the tumor. Collectively, our data along with the literature data suggest the introduction of temozolomide into the therapy regimen not earlier than 8–9 days after treatment of the resection cavity by VV-GMCSF-Lact to reduce the risks of inhibiting the effectiveness of the chemotherapy or virus drug.

We evaluated this hypothesis in an in vivo model. While therapy of U87 MG-bearing mice with TMZ alone has not produced any encouraging results, the VV-GMCSF-Lact has shown significant reductions in tumor volumes, which confirms our existing data [16]. According to the data obtained, it can be concluded that therapy of glioblastoma with the viral drug VV-GMCSF-Lact, both in monotherapy and in combination with TMZ, is significantly more effective than therapy with TMZ alone. Despite the fact that no significant differences in TGI indices between the “VV-GMCSF-Lact”, “VV-GMCSF-Lact+TMZ”, and “TMZ+VV-GMCSF-Lact” groups were found, histological analysis data indicate that the combined therapy is characterized by a more significant effect. Moreover, when VV-GMCSF-Lact is applied before TMZ, it results in a greater degree of destruction of tumor tissue.

Thus, taking into account the data from in vitro and in vivo analysis, the introduction of temozolomide into the treatment regimen should be carried out after handling of the resection field with VV-GMCSF-Lact. In this case, the oncotoxic effect of the drugs will be directed only at the tumor cells remaining after resection, which will significantly diminish the likely side effects of the inflammatory process as a result of virotherapy. On the other hand, this combination will make it possible to decrease the dose of temozolomide in the subsequent course. Eventually, this will not only reduce the risk of relapse but also improve the quality of patients’ lives.

## 4. Materials and Methods

### 4.1. Cell Lines and Patient-Derived Cell Cultures

Glioblastoma U87 MG and U343 MG cell lines and patient-derived BR2.20 and BR5.21 cell cultures were from the cell culture collection of ICBFM SB RAS (Novosibirsk, Russia). Patient-derived cultures were obtained as previously described [16].

### 4.2. Oncolytic Virus

Recombinant VV-GMCSF-Lact was engineered from L-IVP vaccinia virus and has deletions of the viral thymidine kinase (*tk*) and vaccinia growth factor (*vgf*) gene fragments, in the regions of which the genes of human GM-CSF and the oncotoxic protein lactaptin are inserted [13].

### 4.3. Animals

Female SCID mice (6–8 weeks old) were obtained from the SPF-vivarium of the Institute of Cytology and Genetics SB RAS (Novosibirsk, Russia). Mice were kept in the same room within a specific pathogen-free animal facility with a regular 12/12 h light/dark cycle at a constant room temperature of 22  ±  2 °C and relative humidity of approximately 45  ±  15%.

### 4.4. Drugs Preparation

Temozolomide (TMZ) (Sigma-Aldrich, Saint Louis, MO, USA) was prepared in DMSO (Merck, Darmstadt, Germany) with 150 mM TMZ concentration. To evaluate the temozolomide cytotoxic dose, a set of concentrations from 11.25 μM to 840 μM was tested on glioma cells. DMSO final concentration was no more than 0.5%. Lomustine (CCNU) (Abcam, Cambridge, UK) was prepared in EtOH and concentrations from 5 nM to 200 nM were tested. EtOH final concentration was no more than 1.4%. DMSO and EtOH control experiments were performed in order to exclude its toxic effects. Vincristine (VCR) (Sigma-Aldrich, Saint Louis, MO, USA) was dissolved in sterile water with 2 mM VCR concentration. A set of VCR concentrations from 0.05 μM to 200 μM was tested on glioma cells. Procarbazine (PCB) (Abcam, Cambridge, UK) was dissolved in sterile water and tested on glioma cells with concentrations from 0.09 to 1 mg/mL.

VV-GMCSF-Lact, TMZ, VCR, CCNU and PCB at its final concentrations were prepared in OptiMEM medium (Thermo Fisher, New York, NY, USA) with 10% FBS.

### 4.5. Cell Viability Assay

Cell viability was detected using the Deep Blue Cell Viability Kit (BioLegend, San Diego, CA, USA) and determined relative to the viability of the control cells (100%)  ±  standard deviation in three independent experiments. Cytotoxic dose (CD50, the dose at which 50% of cells die) of drugs and the combination index (CI) of drug combination were evaluated using Compusyn software v1.0 (ComboSyn Incorporated, Paramus, NJ, USA).

To assess the cytotoxicity of an individual drug, each reagent (TMZ, VCR, CCNU, and PCB) was added to cells at the concentrations described previously.

To analyze the combined effect of the drugs, VV-GMCSF-Lact and TMZ were added at different time intervals as needed. Concentrations of VV-GMCSF-Lact were equal to CD50 for each cell culture. For the test, concentrations of TMZ were chosen to be less than CD50, CD50, and more than CD50 for each culture. TMZ was added to cells after 12, 24, 36, 48, or 60 h after virus administration. The total time of cell incubation with the drugs was 72 h at 37 °C in an atmosphere of 5% CO_2_.

After incubation with drugs, commercial reagent was added into wells, and the optical density was measured on the spectrophotometer Apollo LB 912 (Berthold Technologies GmbH & Co., KG, Bad Wilbad, Germany) according to the manufacturer’s instructions.

### 4.6. Antitumor Efficacy of VV-GMCSF-Lact Combined with Temozolomide against Subcutaneously Transplanted Human Glioblastoma Cells into SCID Mice

U87 MG cells (3 × 106 cells per mouse, in 100 µL PBS) were subcutaneously transplanted into SCID mice. The tumor volumes (Vt) were measured and calculated using the following Equation (1):

Vt = (a2 × b)/2,(1)

where a is the shorter tumor diameter in mm, and b is the longer tumor diameter in mm.

When the tumor reached 100–120 mm^3^ in size, tumor-bearing mice were treated with drugs according to Table 3.

The tumor growth inhibition (TGI) in all in vivo experiments was calculated using the following Equation (2):

TGI (%) = 1 − (Vt/Vc) × 100 (%),(2)

where Vt is the mean tumor volume of treated mice, and Vc is the mean tumor volume of control animals.

### 4.7. Histology and Morphometry

For the histological study, the tumor specimens were fixed in 10% neutral-buffered formalin (BioVitrum, Moscow, Russia), dehydrated in ascending ethanols and xylols and embedded in HISTOMIX paraffin (BioVitrum, Moscow, Russia). The paraffin sections (5 μm) were sliced on a Microm HM 355S microtome (Thermo Fisher Scientific, Waltham, MA, USA) and stained with hematoxylin and eosin. All the images were examined and scanned using Axiostar Plus microscope equipped with Axiocam MRc5 digital camera (Zeiss, Oberkochen, Germany) at magnifications of ×100, ×200 and ×400.

Mitotic activity in tumor tissue was analyzed by calculating the numerical density (Nv) of mitoses per unit test area. The degree of destructive changes in the tumor node was assessed by calculating the section area occupied by tumor tissue in a state of necrotic decay, including that infiltrated with granulocytes, in relation to the entire section area of the tumor node. During the morphometric study, from 5 to 15 fields of vision were estimated, depending on the condition of the tumor node and the degree of its destruction.

### 4.8. Statistics

Significance in in vitro experiments was determined using a two-tailed Student’s *t*-test. Significance in in vivo experiments was determined using the Mann–Whitney U test.

All error bars represent standard deviation of the mean; *p* < 0.05 was considered to be statistically significant.

## 5. Conclusions

The study of the combined effect of drugs for the tumor treatment in in vitro and in vivo models is very important, as it allows the adjustment of the scheme in further preclinical and clinical studies. In this work, we propose a scheme of combined treatment in which temozolomide can be used not earlier than 8–9 days after VV-GMCSF-Lact, both in further in vivo studies and in clinical practice when confirming the results in animals. The present study lays the foundation for future clinical trials of oncolytic virus VV-GMCSF-Lact and other vaccinia viruses for the treatment of gliomas. Moreover, the effect of joint action with other alkylating agents can be predicted. At the same time, the search for targets involved in the formation of resistance to the combination will make it possible to select targeted drugs for more effective precision therapy.

## Figures and Tables

**Table 1:** VV-GMCSF-Lact cytotoxic activity against glioma cells.

Cell Culture	CD50 *, PFU/Cell
U87 MG	0.1 ± 0.028
U343 MG	0.06 ± 0.008
BR2.20	0.0002 ± 0.000007
BR5.21	0.5 ± 0.018

* CD50—cytotoxic dose causing death of 50% cells.

**Table 2:** Sensitivity of glioma cells to temozolomide, lomustine, and vincristine.

Drug	Cell Culture			
	U87 MG	U343 MG	BR5.21	BR2.20
	CD50 *			
Temozolomide, µM	437.3 ± 2.1	25.3 ± 0.9	297.2 ± 1.3	408.2 ± 2.4
Vincristine, nM	1 ± 0.2	0.3 ± 0.04	135.1 ± 1.1	11.3 ± 0.7
Lomustine, nM	21.4 ± 1.4	17.5 ± 0.7	86 ± 2.2	n/a

* CD50—cytotoxic dose causing death of 50% cells.

**Table 3:** Scheme of treatment of tumor-bearing mice.

Group (6 Mice per Group)	Scheme
VV-GMCSF-Lact	The virus was administered intratumorally three times at an interval of 7 days with a dose of 10^7^ PFU/mouse.
TMZ	TMZ was administered intraperitoneally four times over 4 days with a dose of 7.5 mg/kg.
VV-GMCSF-Lact+TMZ	The virus was administered intratumorally one time with a dose of 10^7^ PFU/mouse. After 8 days, mice were treated with four intraperitoneal injections of TMZ (7.5 mg/kg) over 4 days.
TMZ+VV-GMCSF-Lact	TMZ was administered intraperitoneally four times over 4 days with a dose of 7.5 mg/kg. After 72 h, mice were treated with intratumoral injections of the virus three times at an interval of 7 days with a dose of 10^7^ PFU/mouse.
control	Mice were not treated.

**Table 4:** TGI indices in the treatment of subcutaneously transplanted glioblastoma U87 MG with VV-GMCSF-Lact, temozolomide, or its combination.

Group	TGI *, %
VV-GMCSF-Lact	93
TMZ+VV-GMCSF-Lact	96
VV-GMCSF-Lact+TMZ	93
TMZ	33

* TGI—tumor growth inhibition index.

**Table 5:** Structural changes in U87 MG glioblastoma tissue without treatment and after administration of temozolomide, VV-GMCSF-Lact, or their combination.

	Control	TMZ	VV	VV + TMZ	TMZ + VV
Mitosis, Nv	5.5 ± 0.7	3.6 ± 0.6	0.6 ± 0.4 **	0	0
Destructive changes, %	52.1 ± 9.5	62.5 ± 12	74.5 ± 9.8	93.6 ± 3.6 *	66.8 ± 2.5

Differences are statistically significant from control at *p* ≤ 0.05 (*) and *p* ≤ 0.001 (**).

## Data Availability

Data are contained within the article and Supplementary Materials.

## Supplementary Materials

The following supporting information can be downloaded at: https://www.mdpi.com/article/10.3390/ijms25084244/s1.
