# Supplementary material for: The Recombinant Oncolytic Virus VV-GMCSF-Lact and Chemotherapy Drugs against Human Glioma
Source: Int J Mol Sci. 2024 Apr 11;25(8):4244. doi: 10.3390/ijms25084244 (PMC11049884; doi:10.3390/ijms25084244)
Supplement: Supplementary file 1 [file ijms-25-04244-s001.zip › ijms-2941431-supplementary.pdf]

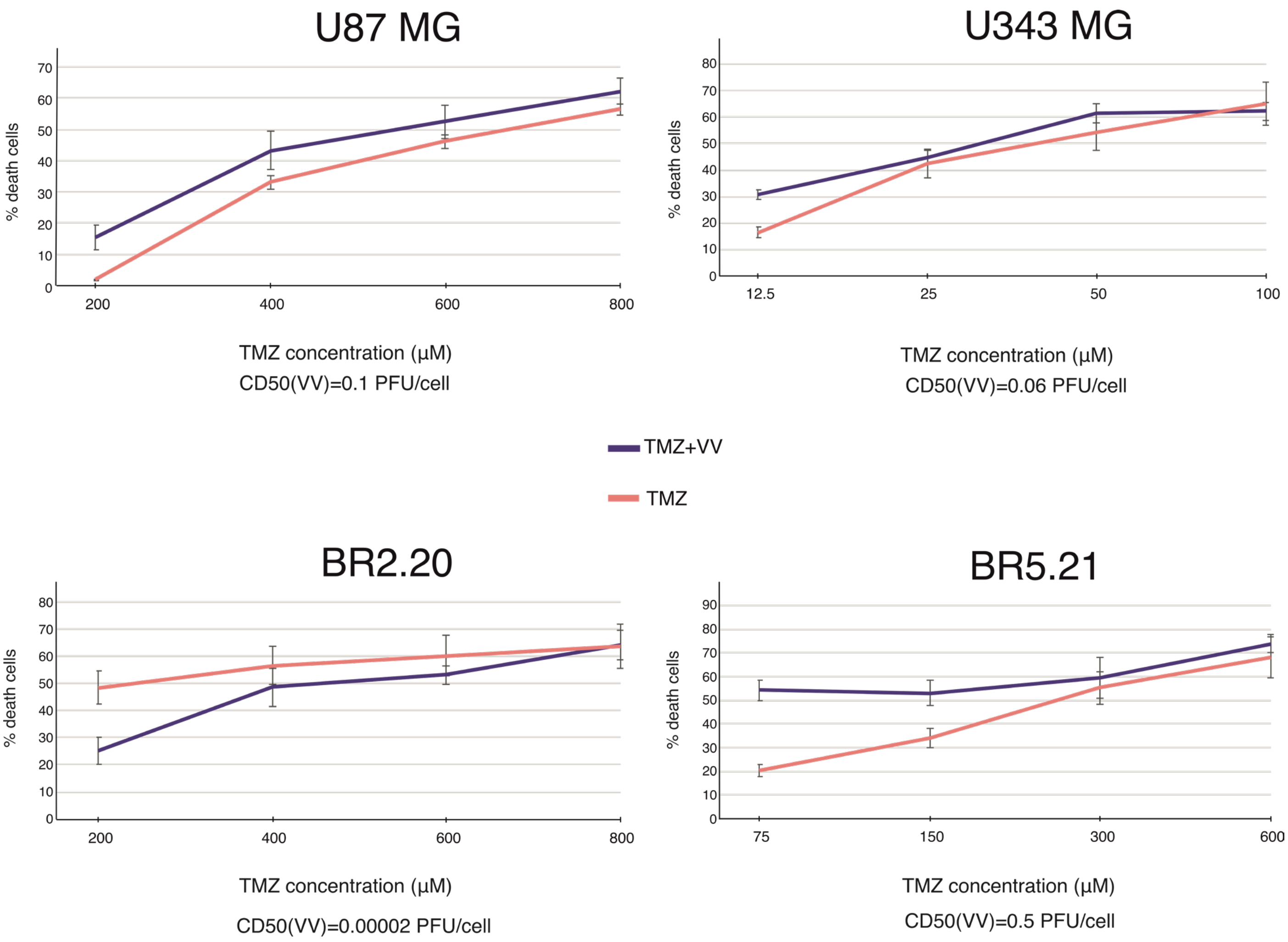

Figure S1. Changes in the viability of human glioma cells under the action of temozolomide followed by the addition of VV-GMCSF-Lact. VV-GMCSF-Lact was added to the cells after 24 h incubation with TMZ. CD50(VV)—multiplicity of virus infection, in which 50% of cells die; VV—VV-GMCSF-Lact; TMZ—temozolomide.
